# Endotracheal Tube Size Estimation in Paediatric Patients: A Head-to-head Comparison of Accuracy Between Ultrasonography and Age-based Formula

**DOI:** 10.4274/TJAR.2025.241822

**Published:** 2025-03-21

**Authors:** Archan Jayantbhai Bhut, Kalyani Nilesh Patil, Sarita Swami

**Affiliations:** 1 Bharati Hospital Bharati Vidyapeeth (Deemed to be University) Medical College, Clinic of Anaesthesiology and Reanimation, Pune, India

**Keywords:** Airway management, cricoid cartilage, intubation, paediatric anaesthesia, ultrasonography

## Abstract

**Objective:**

In the paediatric population, the selection of an appropriately sized endotracheal tube (ETT) is extremely important not only to ensure adequate ventilation but also to avoid post-extubation stridor and stenosis. Conventionally, formulas based on age, height, or weight are used to determine the most appropriate size. In this study, we compared ultrasonography (USG) and age-based formula for predicting the best microcuff ETT size in paediatric patients aged 1-5 years.

**Methods:**

One hundred eighteen patients, aged 1 to 5 years, with American Society of Anesthesiologists, classifications of I or II, were included. After standard general anaesthesia protocols, the subglottic diameter was assessed by USG. Intubation was performed using ETT size according to age-based formula. The best clinical fit was determined after the leak test. The internal and external diameters of the ETTs were predicted by both methods and correlated with the best-fit ETT sizes used during the procedures using Pearson's correlation. Cohen’s kappa was used for statistical agreement between two methods.

**Results:**

USG had a significantly higher correlation with the best-fit model as compared to the age-based formula, with 99.2% and 77.1% agreement rates. The best-fit ETT showed a better correlation with the USG-guided estimate (r = 0.994, *P* < 0.001). The Cohen’s Kappa value of 0.986 showed a statistically significantly higher agreement between USG-guided estimate and best-fit ETT.

**Conclusion:**

USG-guided estimation of subglottic diameter is a better predictor for optimally sized microcuff ETT than the age-based formula in the paediatric age group of 1-5 years. 22% of tube changes could have been prevented with ultrasound-guidance as a primary approach for estimating ETT size.

**Main Points**

• Our study aims to compare the effectiveness of ultrasonography (USG)-based estimation with age-based formula estimation for determining the appropriate endotracheal tube (ETT) size in children aged 1 to 5 years.

• The uniqueness of our study is a larger sample size, younger age group with a mean of 2.79±1.14 years, the use of USG measurement done after the paralytic agent and cessation of ventilation, and the use of a microcuff tube.

• 22% of ETT changes could have been prevented with ultrasound-guidance as a primary approach for estimating ETT size.

## Introduction

General anaesthesia is the cornerstone of anaesthetic care for paediatric patients. Prior to performing tracheal intubation, it is essential to possess a thorough understanding of airway anatomy. Paediatric airway anatomy differs significantly from that of adults.^1^ In paediatric anaesthesia, the use of precise endotracheal tube (ETT) size is extremely crucial for irst attempt success at intubation. Failure in selecting the appropriate ETT size can result in inadequate ventilation and the nability to maintain general inhalational anaesthesia.^2^

Recent evidence further suggests that, even though the microcuff tubes are marketed as safe, their cuff design may be associated with cuff-induced injury to the vocal cords.^3^ If the ETT size is bigger, it can significantly cause friction and compression on the tracheal mucosa, leading to mucosal ischemia and airway edema. This insult can subsequently result in post-extubation complications, which can be subglottic stenosis or stridor.^4^ Conversely, using an ETT that is too small can increase the flow resistance, leading to inadequate ventilation and to higher chances of aspiration.^5^ Additionally, a leak from an undersized ETT can result in the release of anaesthetic gases into the environment.

To overcome these issues at both ends, various formulas were derived and studied to choose the perfect size of ETT based on age, weight, and height.^6, 7, 8^ Age-based formulas derived by Cole^9^ and Motoyama^10^, which have been in clinical use for over half a century, calculate the internal diameter (ID) of the uncuffed tube.

However, it’s important to note that the ETT dimensions vary over a wide range among the manufacturers. The ETT with the same ID can have different outer diameters (OD) depending on manufactures, potentially resulting in misleading calculations. None of these calculations demonstrate optimal effectiveness, and moreover, they may not be applicable to all ethnic populations worldwide.

The modernization of the healthcare system has led to the increasing popularity of ultrasonography (USG) in perioperative airway management. USG serves various purposes in airway management, such as identifying structures, ensuring proper positioning, and selecting the correct ETT size.^11, 12^

Although previous studies have explored the feasibility of USG for assessing subglottic diameter as a predictor of ETT size, there are very few studies estimating the size of the microcuff tubes, which are now a standard of care in paediatric anaesthesia.^13^ Therefore, we designed our study with the aim to compare USG-guided and age-based formula estimation of ETT size for paediatric patients and to assess if the USG method provides a better estimation. We have further studied the frequency of reintubations and the time taken by both methods.

## Methods

After approval from the Bharati Vidyapeeth (Deemed to be University) Medical College, Institutional Research Ethics Committee (approval no.: BVDUMC/IEC/80, date: 12.08.2022), the Clinical Trials Registry-India (CTRI) registration (CTRI/2023/06/054327), and written informed consent from guardians, a prospective observational study was conducted in a tertiary care hospital. The inclusion criteria were 118 patients, posted for elective surgeries needing general anaesthesia, aged 1 to 5 years, with American Society of Anesthesiologists classifications of I or II.

Patients with pre-existing laryngeal or pharyngeal pathology, airway deformity, presence of a scar on the neck, ulcer or mass, upper respiratory tract infection were excluded.

The sample size was calculated with reference to Gehlaut et al.^14^

$\mathrm{Sample}\;\mathrm{size}\;(n)\;=\frac{2z^{2}(1-\alpha /2)\times {\left(S\right)}^{2}}{d^{2}}$

z (1-α/2) = 1.96 (standard normal value at 5% level of significance).

S = 0.781 pooled standard deviation (SD) value from previous reference study.

d = 0.2 allowable error (absolute precision).

As per standard protocols, all patients were nil per oral on the day of surgery. The standard anaesthesia workstation check protocol was followed before each anaesthetic to ensure safety and proper functioning of equipment.

The patient was then escorted to the operation theatre, where standard monitoring was initiated. This includes continuous monitoring of heart rate by electrocardiography, saturation level of oxygen by pulse oximetry, blood pressure by non-invasive blood pressure monitoring, temperature monitoring, as well as the end-tidal CO_2_.

Standard general anaesthesia protocols were followed in all patients as follows:

• Premedication: Intravenous (IV) Inj. glycopyrrolate 4 µg kg^-1^ and midazolam 0.05 mg kg^-1^

• Preinduction: Inj. fentanyl 2 µg kg^-1^ IV,

Patients were pre-oxygenated with 100% oxygen for a span of 3 minutes.

• Induction: Inj. propofol 2-2.5 mg kg^-1^ IV, titrated to effect.

• A loading dose of the neuromuscular blocking agent: Inj. atracurium 0.5 mg kg^-1^ intravenously was given for facilitating intubation. Ventilation was continued for another 4 minutes.

**Figure 1:**
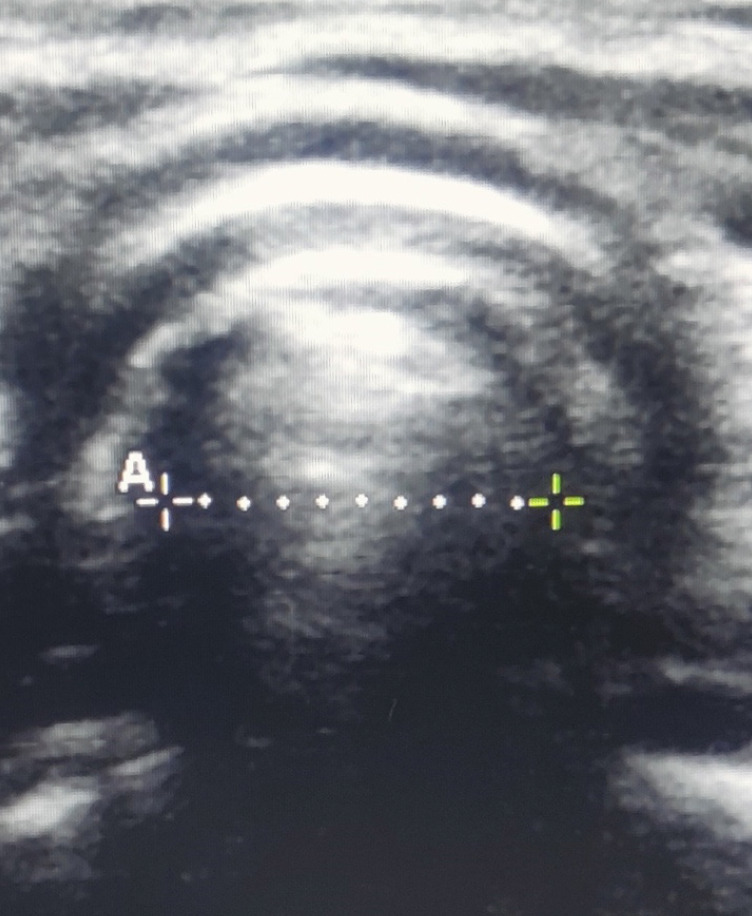
USG view of cricoid arch and air-column. Cricoid cartilage is a round hypoechoic structure with hyperechoic edges. The air column (dotted line) appeared hyperechoic and created a posterior acoustic shadow. The mucosa-air interface, a hypoechoic edge, was easily recognized. The dotted line represents the measured air-column width. USG, ultrasonography.

After giving muscle relaxant and before intubation, in sniffing position with continued mask ventilation, an ultrasound scan was done using a linear probe (6-13 Hz frequency) to identify the level of cricoid cartilage. The ventilation was momentarily ceased, and the timer was started simultaneously until we obtained an optimal view at the level of cricoid (subglottic tracheal diameter). The screen was then frozen and ventilation resumed. The distance (air column width) was calculated on the “frozen” USG screen using the caliper function of the ultrasound machine. This diameter was used as a surrogate of the OD of the microcuff ETT. Figure 1, dotted line shows the OD. Now, the corresponding ID was determined from the diameters marked on the surface of the different microcuff tubes. With the OD by USG noted as 4.3, 5.0, 5.6, 6.3, 6.77 millimetres (mm) the corresponding ID as 3.0, 3.5, 4.0, 4.5, 5.0 was respectively noted.

In all patients, endotracheal intubations were performed with the micro-cuffed ETT of size, calculated by the age-based formula.

ID (mm) = 0.25×(age in years)+3.5

The time required for the calculation of the age-based formula was measured by starting the timer once the calculator was turned on and stopping it upon the completion of the calculation. The timer stopped following the last answer regarding the tube size.

The microcuff tubes used were from the same manufacturer (Halyard). Endotracheal intubations were then performed with the size determined by age-based formula; and after performing the leak test, the actual ETT used clinically was labeled as “best-fit” and noted.

The leak test technique was applied to determine the optimal size. This method uses leak pressure (LP) to determine the ideal ETT size for paediatric patients. LP, which is the pressure at which air escapes around the tube, was measured by placing a stethoscope over the suprasternal notch while monitoring the manometer on the ventilator to detect the pressure at which the leak was audible. The ETT size was considered optimal when the tracheal leak occurred at an inflation pressure between 10 and 25 cmH_2_O. If no air leak was detected at airway pressures of 25 cmH_2_O, tube was replaced with one that was 0.5 mm smaller. Conversely, if the leak was detected at a pressure lower than 10 cmH_2_O, a 0.5 mm larger tube was selected.^15, 16, 17^ Due to the use of a cuffed ETT, the LP measurement was evaluated prior to cuff inflation.

### Statistical Analysis

The data for normally distributed continuous variables were presented as mean±SD, while categorical variables were expressed as frequencies n (% of cases). A paired t-test was applied to compare the means of the continuous variables (i.e., ETT sizes and time taken to calculate ETT size). Pearson’s correlation was used for correlation analysis. The normality assumption was assessed before applying the t-test and Pearson’s correlation analysis to the study variables. Cohen’s Kappa is used for statistical agreement between two methods. To visualize the statistically significant difference more clearly, these results were shown in both tabular and graphical formats.

The complete study data were statistically analysed using the Statistical Package for Social Sciences (SPSS version 24.0, IBM Corp., Armonk, NY, USA) for Microsoft Windows. The *p *values <0.05 were considered to be statistically significant for the entire study.

## Results

**Figure 2:**
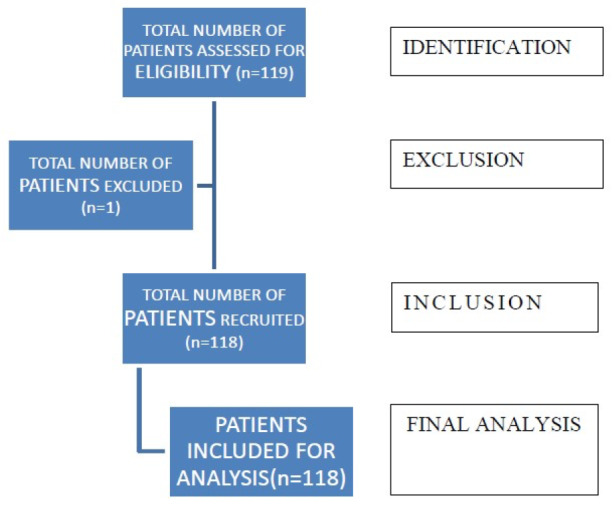
Strobe’s flow graph.

The total number of patients assessed for eligibility was 119; 1 patient was excluded because of an unanticipated difficult airway, and finally the study involved 118 paediatric patients aged 1-5 years and undergoing surgery under general anaesthesia, as demonstrated in Strobe’s chart (Figure 2). The mean age of patients included was 2.74±1.37 years. 46.6% of patients were between the age of 1-2 years (55 patients) and 53.4% of patients were between age of 3-5 years (63 patients). They comprised 88 male and 30 female patients. Minimum weight of 8 kg and maximum of 22 kg with mean 12.15±2.94 kg. The mean weight for the age group of 1-2 years and 3-5 years was 10±1.5 kg and 13.98±2.63 kg, respectively.

The mean±SD of ETT size by best-fit and USG-guided method was 4.28±0.41 mm and 4.29±0.42 mm, respectively. For the age group of 1-2 years, the ETT size by best-fit and USG-guided method was 4.06±0.30 mm and 4.07±0.32 mm, respectively. On the other hand, for patients aged 3-5 years, the ETT size by both the best-fit and USG-guided method was 4.47±0.40 mm.

On a paired t-test, the mean best-fit ETT size did not differ significantly from the mean ETT by USG-guided method, (*p* > 0.05). The mean±SD of ETT size by best-fit and age-based estimation was 4.28±0.41 mm and 4.22±0.38 mm, respectively. In a paired t-test, the mean best-fit ETT size is significantly higher than the mean ETT size based on age estimation (*p* < 0.05). The mean percentage change or absolute deviation in ETT size by age-based estimation with reference to the best-fit ETT size was 2.88%. The mean percentage change or absolute deviation in ETT size by the USG-guided method compared to the best-fit ETT size was 0.094%. Table 1 shows the paired comparison of ETT size (ID) by different methods.

Out of 118 cases, 117 (99.2%) had a perfectly matched size, 1 (0.8%) had an overestimated size, and none underestimated the ETT size by the USG-guided method against the best-fit ETT size. Out of 118 cases, 91 (77.1%) had perfectly matched size, 8 (6.8%) had the overestimated size, and 19 (16.1%) had the underestimated ETT size by age-based estimation against the best-fit ETT size.

**Figure 3:**
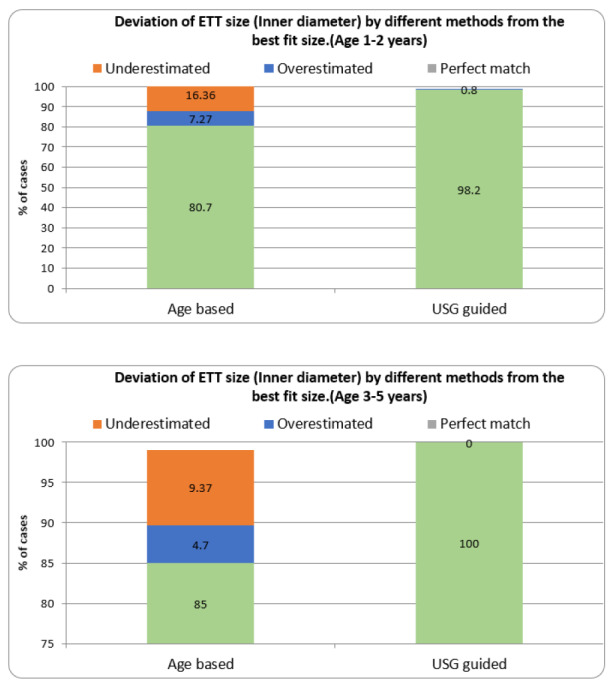
Deviation of ETT size by different methods from the best-fit size. USG, ultrasonography; ETT, endotracheal tube.

For 1-2 years, out of 55 cases, 54 (98.2%) had a perfectly matched size, 1 (0.8%) had an overestimated size, and none had underestimated the ETT size by the USG-guided method against the best-fit ETT size. On the other hand, 42 patients (80.7%) had a perfectly matched best-fit size, 4 (7.27%) had an overestimated size, and 9 (16.36%) had an underestimated ETT size by age-based estimation against the best-fit ETT size. Out of 63 patients aged 3-5 years, the ETT size determined by USG correlated 100%. While on other hand for age based estimation method only 54 patients (85%) had a perfect matched size with best-fit, 3 (4.7%) had an overestimated size and 6 (9.37%) had an underestimated the ETT size (Figure 3).

The distribution of ETT size by USG-guided method is significantly associated with the best-fit ETT size (*p* < 0.05) with a Cohen’s Kappa value of 0.986. There is a proven statistically significant and higher-to-perfect agreement with regard to USG-guided ETT size and the best-fit ETT size in our study group (Table 2). The distribution of ETT size by age-based and best-fit ETT shows statistically moderate agreement, indicated by a Cohen’s Kappa value of 0.601 (Table 3).

**Figure 4:**
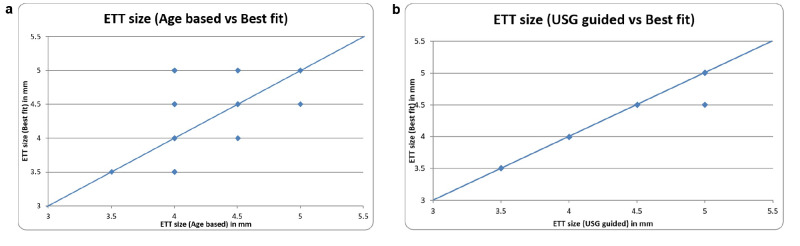
a) Scatter diagram showing correlation between ETT size (Inner diameter) by age-based estimation and best-fit ETT size. b) Scatter diagram showing correlation between ETT size (Inner diameter) by USG guided method and best-fit ETT size. USG, ultrasonography; ETT, endotracheal tube.

As per Pearson’s correlation analysis, ETT size by the USG-guided method showed a statistically strong positive correlation with the best-fit ETT size with correlation coefficient r = 0.994, (*p* <0.001). On the contrary, ETT size by age-based estimation showed a statistically moderate correlation with the best-fit ETT size, with a correlation coefficient r = 0.772 (*p* < 0.001) (Figure 4a, b).

The mean±SD time taken to calculate ETT size by age-based estimation and USG-guided estimation was 6.63±0.98 seconds and 13.00±1.33 seconds, respectively. On a paired t-test, the mean time taken to calculate ETT size by the USG-guided method is significantly longer than the mean time taken to calculate ETT size by age-based estimation (*P* < 0.05). Out of 118 patients, 26 needed their ETT replaced, which accounts for 22% of the total participants.

## Discussion

Securing the paediatric airway with an appropriately sized ETT is both extremely important and daunting. If the tube is oversized or the cuff overinflated, it may damage the tracheal mucosa, leading to airway oedema, post-extubation stridor, subglottic stenosis, or cartilaginous ischemia. On the other hand, if the tube is too small, it will increase the risk of aspiration, occlusion, increase the resistance to airflow, resulting in insufficient ventilation and make the monitoring of end tidal gases unreliable.^5^ Moreover, the correlation between age, height, weight, body surface area, and tracheal shape or size is poor, and hence the formulae may not accurately estimate the tube size.^18^

The tube exchanges and repeated airway instrumentation result in subsequent oedema or trauma leading to complications and long-term sequelae in children, more clinically significant than in adults, which makes it even more pertinent to intubate them with the appropriately sized tube at the first attempt. A reliable way to estimate the variable is important. Ultrasound has proven to be a safe, reliable, non-invasive, and point-of-care method to assess the airway diameter. These attributes make it a feasible tool in the paediatric cohort.^12^

Similar to our study, Gupta et al.^5^ in their study on 112 patients, in the age group of 3-18 years, have shown a higher correlation between clinically used ETTs and predetermined ETTs by USG than the predicted ETT by age and height-based formulas. They have further validated the reliability of an ultrasound for measuring the subglottic diameter and hence avoiding intubation-related complications.

Hatfield and Bodenham^19^, in their report on the feasibility of USG in assessing the subglottic diameter, have shown a positive correlation between USG and magnetic resonance image measurements of the transverse subglottic diameter. Lakhal et al.^12^ have also concluded that USG is a reliable, non-invasive, bedside tool in assessing the smallest transverse diameter of the cricoid lumen.

Laksono et al.^20^, in their study on Indonesian paediatric patients, compared the accuracy of uncuffed ETT size estimation using USG, the body length formula, and the left-hand 5^th^ fingernail width method, and observed that USG had the highest accuracy at 92%, while the body length formula had the lowest at 64% in estimating uncuffed ETT size. USG is seven times more likely to estimate the precise uncuffed ETT size than the body length formula and five times more likely than the left-hand 5^th^ fingernail width.

Bae et al.^21^ also concluded that ultrasound was a better means for estimating ETT size in paediatric patients than the age-based formula. Both these studies, however, estimated the accuracy in estimating the size of uncuffed ETT, whereas we have used microcuffed tubes in our study.

Umbarkar and Vaishnav^22^ concluded that the rate of differences between ultrasonographically determined ETT size and correctly sized ETT with a maximum allowed deviation of ≤0.3 mm was 89.18% for uncuffed tubes and 86.95% for cuffed tubes. They further concluded that, as compared to physical indices-based formulae, the USG predicted the appropriate ETT size (*p* < 0.05) better for both cuffed and uncuffed tubes.

Similar to our study, Pillai et al.^23^ in their study, observed that age‑based formula showed poor correlation (27.5%) compared to ultrasound (87.8%) in predicting the best‑fit ETT. They concluded that paediatric patients with congenital heart disease required a larger ETT as compared to that predicted by age-based formula, and ultrasound is a safe and accurate method for estimating the best-fit ETT size in the paediatric cardiac population.

Similarly, Singh et al.^24^ used Pearson’s correlation and concluded that there was only a moderate correlation of the best-fit ETT with the estimated ETT size by age-based formula (r = 0.743), body length-based formula (r = 0.683), right little finger-based formula (r = 0.587), left little finger-based formula (r = 0.587) and multivariate formula (r = 0.741). However, the correlation of best-fit ETT with USG estimated ETT size was strong (r = 0.943). Similar to our study, all the radiological measurements in this study were performed by an experienced and trained anaesthesiologist.

In addition, we have also used other ways to prove the significance; 0.986 as the Cohen’s Kappa value showed a statistically significant near perfect agreement between USG-guided ETT size and best-fit ETT size in the study group.

Recent studies by Gunjan et al.^25^, Putra et al.^26^, Ekor et al.^27^, Gooty et al.^28^, and Zengh et al.^29^ have all concluded that USG, in addition to being safe and non-invasive, also offers greater accuracy in estimating the best-fit ETT as compared to the conventional methods.

The findings of Bae et al.^21^ partly concur with those of our study. They assessed the usefulness of USG in determining uncuffed tracheal tube sizes for paediatric patients. They concluded that USG could provide a valuable alternative to the conventional age-based formula for selecting an appropriate ETT size in paediatric patients. However, despite the use of USG in their study, only 60% of cases achieved the correct tube size. The reason for this, according to the authors, could be the variations in the external diameter of tracheal tubes according to the manufacturer. In our study, we found that USG-guided ETT size has 99.2% agreement with the best-fit ETT. This difference could be attributable to the fact that in our study we exclusively used microcuffed tubes by the same manufacturer.^21^

In our study, we have further compared the time required for calculation of ETT size by age-based formula as well as by ultrasound. The time taken to calculate ETT by age-based formula was around 7 seconds, whereas with ultrasound it was around 14 seconds.

Though the time was less with the age-based formula, out of 118 patients, 26 patients needed the tube change after the first intubation. These 22% ETT changes would have been avoided if we had used USG guided method as our primary tool for ETT size estimation. This high incidence of tube exchange negates any advantage of faster calculation with the age-based formula. This finding is consistent with the results of Schramm et al.^30^, who found that the initial choice of ETT was incorrect in 23 out of 50 patients (46%), requiring the insertion of an alternative tube. They concluded that USG aids in selecting the correct ETT size in paediatric patients and may help decrease the need for reintubations.^23, 30^

### Study Limitations

Our study has a few limitations. We included patients only from a single center, and larger, multicentric studies are recommended.

The cessation of ventilation is required for accurate calculation of ETT size in paediatric patients. However, the saturation was maintained in all patients and patients were ventilated soon after freezing the screen for further calculation. The avoidance of tube exchanges with ultrasonographic assessment is a definite advantage. Also, in our study, we have not included postoperative bronchoscopy to visualize if any anatomical injuries are caused due to a “non-fitting” ETT.

However, our study is unique in various aspects. We studied a larger sample size, and a younger age group of patients with a mean 2.79±1.14 years, using microcuff tube instead of uncuffed or regular cuffed tubes. Also, for avoiding miscalculation, USG measurement is done after paralytic agent and momentary cessation of ventilation. All the USG measurements and intubations were performed by an experienced anaesthesiologist trained in paediatric anaesthesia and ultrasonography. We have gone further ahead and compared the time required for calculating the best-fit ETT with an age-based formula and ultrasound which has important clinical implications in a paediatric patients. All these factors make our study stand out as compared to the previous studies.

## Conclusion

To conclude, USG-guided subglottic diameter is a better predictor of the optimal size of the microcuff ETT than the age-based formula. USG has proved to be a non-invasive, safe, and reliable tool. We recommend the use of USG on a regular basis as a primary tool for ETT size determination in paediatric patients to avoid multiple laryngoscopies attempts and change of ETT.

## Ethics

**Ethics Committee Approval:** Ethical approval was obtained from the Bharati Vidyapeeth (Deemed to be University) Medical College, Institutional Research Ethics Committee (approval no.: BVDUMC/IEC/80, date: 12.08.2022).

**Informed Consent:** Written informed consent was obtained from guardians.

## Figures and Tables

**Table 1. Paired Comparison of ETT Size (Inner Diameter) by Different Methods:** 

-	**ETT size (mm)**	
Method	Mean±SD	Median (Min.-Max.)
Best-fit	4.28±0.41	4.00 (3.5-5.0)
Age-based	4.22±0.38	4.00 (3.5-5.0)
USG-guided	4.29±0.42	4.00 (3.5-5.0)
Age based vs best-fit (% deviation)	2.88%	-
USG guided vs best-fit (% deviation)	0.094%	-
*P *value (Paired comparisons)	-	-
Best-fit vs Age-based	0.019*	-
Best-fit vs USG-based	0.319NS	-

*P *value by paired t-test;* P *< 0.05 is considered to be statistically significant; *P < 0.05.

NS, statistically non-significant; USG, ultrasonography; ETT, endotracheal tube; SD, standard deviation; Min.-Max., minimum-maximum.

**Table 2. The Statistical Agreement Between ETT Size by USG Guided Method and Best-fit ETT Size:** 

-	**ETT Size in mm (Best-fit)**								-	
-	**3.5**		**4.0**		**4.5**		**5.0**		**Total**	
ETT size in mm (USG guided)	n	%	n	%	n	%	n	%	n	%
3.5	5	100.0	0	0.0	0	0.0	0	0.0	5	4.2
4.0	0	0.0	62	100.0	0	0.0	0	0.0	62	52.5
4.5	0	0.0	0	0.0	29	96.7	0	0.0	29	24.6
5.0	0	0.0	0	0.0	1	3.3	21	100.0	22	18.6
Total	5	100.0	62	100.0	30	100.0	21	100.0	118	100.0
Cohen’s Kappa value = 0.986, *P*=0.001***										

*P* value by chi-square test. Cohen’s Kappa for statistical agreement between two methods. *P* < 0.05 is considered to be statistically significant. ***P < 0.001.

ETT, endotracheal tube; USG, ultrasonography.

**Table 3. The Statistical Agreement Between ETT Size by Age-Based Estimation and Best-fit ETT Size:** 

-	**ETT size in mm (Best-fit)**								-	
-	**3.5**		**4.0**		**4.5**		**5.0**		**Total**	
ETT size in mm (Age based)	n	%	n	%	n	%	n	%	n	%
3.5	1	20.0	0	0.0	0	0.0	0	0.0	1	0.8
4.0	4	80.0	60	96.8	14	46.7	3	14.3	81	68.6
4.5	0	0.0	2	3.2	14	46.7	2	9.5	18	15.3
5.0	0	0.0	0	0.0	2	6.6	16	76.2	18	15.3
Total	5	100.0	62	100.0	30	100.0	21	100.0	118	100.0
Cohen’s Kappa value = 0.601, *P*=0.001***										

*P* value by chi-square test; Cohen’s Kappa for statistical agreement between two methods.* P* < 0.05 is considered as statistically significant; ***P < 0.001.

ETT, endotracheal tube.
